# Drop-off-reinitiation at the amino termini of nascent peptides and its regulation by IF3, EF-G, and RRF

**DOI:** 10.1261/rna.079447.122

**Published:** 2023-05

**Authors:** Takayuki Katoh, Hiroaki Suga

**Affiliations:** Department of Chemistry, Graduate School of Science, The University of Tokyo, Bunkyo-ku, Tokyo 113-0033, Japan

**Keywords:** drop-off-reinitiation, initiation complex, initiator tRNA, IF3, EF-G, RRF

## Abstract

In translation initiation in prokaryotes, IF3 recognizes the interaction between the initiator codon of mRNA and the anticodon of fMet-tRNA^ini^ and then relocates the fMet-tRNA^ini^ to an active position. Here, we have surveyed 328 codon–anticodon combinations for the preference of IF3. At the first and second base of the codon, only Watson–Crick base pairs are tolerated. At the third base, stronger base pairs, for example, Watson–Crick, are more preferred, but other types of base pairs, for example, G/U wobble, are also tolerated; weaker base pairs are excluded by IF3. When the codon–anticodon combinations are unfavorable for IF3 or the concentration of IF3 is too low to recognize any codon–anticodon combinations, IF3 fails to set the P-site fMet-tRNA^ini^ at the active position and causes its drop-off from the ribosome. Thereby, translation reinitiation occurs from the second aminoacyl-tRNA at the A site to yield a truncated peptide lacking the amino-terminal fMet. We refer to this event as the amino-terminal drop-off-reinitiation. We also showed that EF-G and RRF are involved in disassembling such an aberrant ribosome complex bearing inactive fMet-tRNA^ini^. Thereby EF-G and RRF are able to exclude unfavorable codon–anticodon combinations with weaker base pairs and alleviate the amino-terminal drop-off-reinitiation.

## INTRODUCTION

During ribosomal elongation of nascent peptides, consecutive incorporation of less reactive amino acids, such as proline (Pro) and d-amino acids, makes peptidyl transfer inefficient and thereby ribosome often stalls (Muto and Ito 2008; Pavlov et al. 2008; Doerfel et al. 2013; Fujino et al. 2013; Peil et al. 2013; Ude et al. 2013; Starosta et al. 2014; Huter et al. 2017). Then, EF-G triggers mistranslocation of P-site peptidyl-tRNA and A-site aminoacyl-tRNA toward the E and P sites, respectively, prior to completion of peptidyl transfer (Tajima et al. 2022). The E-site peptidyl-tRNA eventually drops off from the ribosome, and the P-site aminoacyl-tRNA allows for reinitiation of translation, leading to synthesis of a truncated peptide lacking the amino-terminal region, referred to as the reinitiated peptide (RiP) (Kang and Suga 2011; Fujino et al. 2013; Tajima et al. 2022). This event is called drop-off-reinitiation.

Here, we have investigated whether or not the drop-off-reinitiation event also occurs at the amino terminus of nascent peptides when the initiator tRNA^ini^ is located at the P site. We refer to this event specifically as the “amino-terminal (or N-terminal)” drop-off-reinitiation to discriminate from the drop-off-reinitiation in elongation. In the canonical elongation event, peptidyl-tRNA is translocated from A site to P site by EF-G and eventually placed at the active position to induce peptidyl transfer. On the other hand, in the initiation event, the initiator fMet-tRNA^ini^ is directly recruited to the P site of the 30S initiation complex (30S IC) and relocated to the P/I position by IF2 in the presence of IF1 and IF3 (Simonetti et al. 2008; Hussain et al. 2016; Kaledhonkar et al. 2019). Then, dissociation of IF3 and association of 50S subunit to the 30S IC occur to form 70S IC, and then IF1 and IF2 are released from the 70S IC to give a 70S elongation complex (EC) with a fully active fMet-tRNA^ini^ at the P/P position. Therefore, the mechanisms how the P-site peptidyl-tRNA is placed at the active site are completely different between the elongation and initiation events. Reactivity and drop-off susceptibility of the P-site peptidyl-tRNAs should also be differently regulated by different mechanisms. Although we previously revealed that EF-G enhances the drop-off-reinitiation frequency in elongation, the frequency of the amino-terminal drop-off-reinitiation might be regulated differently by other translation factors. For instance, it is known that IF3 destabilizes the binding of initiator tRNA to the 30S subunit (Antoun et al. 2006).

In prokaryotes, the canonical AUG codon is usually utilized for initiation and decoded by the CAU anticodon of tRNA^ini^. However, some non-AUG initiator codons, such as GUG and UUG, have also been reported to date (Blattner et al. 1997; Tikole and Sankararamakrishnan 2006; Villegas and Kropinski 2008; Hecht et al. 2017; Roy et al. 2018; Cao and Slavoff 2020). It is known that IF3 recognizes the codon–anticodon interaction between AUG and CAU and then places the fMet-tRNA^ini^ at the active position (Hussain et al. 2016). Although some non-AUG initiator codons are reportedly recognized by IF3 to activate fMet-tRNA^ini^ (Sussman et al. 1996; Grigoriadou et al. 2007), comprehensive analysis for the codon–anticodon combinations tolerated by IF3 has not been performed to date to the best of our knowledge. Therefore, here we have aimed at performing such a comprehensive study to reveal the preference of IF3 for 328 codon–anticodon combinations and its effect on the frequency of the amino-terminal drop-off-reinitiation. We have also evaluated the effect of EF-G and RRF on the drop-off-reinitiation considering their involvement in peptidyl-tRNA drop-off (Gong et al. 2007; Singh et al. 2008; Tajima et al. 2022). As the initiator substrate, not only fMet but also *N*-acetyl-l-proline (AcPro) were tested because we can expect higher drop-off susceptibility for Pro (or AcPro) due to its significantly slow rate of peptide bond formation (Sussman et al. 1996; Muto and Ito 2008; Pavlov et al. 2008; Doerfel et al. 2013; Peil et al. 2013; Ude et al. 2013; Starosta et al. 2014; Huter et al. 2017; Tajima et al. 2022). By optimizing the translation conditions such as the concentrations of IF3, EF-G, and RRF, we could efficiently suppress drop-off-reinitiation even for the inefficient AcPro incorporation. Thereby, we can expect efficient ribosomal expression of exotic peptides with inefficient initiator substrates such as AcPro.

## RESULTS

### Drop-off-reinitiation at the amino terminus of nascent peptides

**FIGURE 1.**
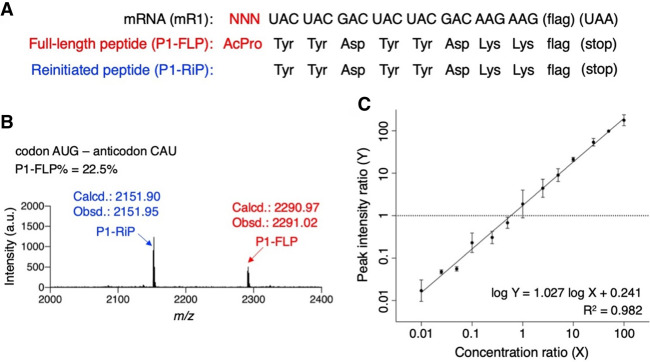
In vitro translation of a model peptide introducing *N*-acetyl-l-proline at the amino terminus. (*A*) Sequences of mRNA, mR1, and the corresponding peptide sequences P1-FLP and P1-RiP. P1-FLP is a full-length peptide bearing *N*-acetyl-l-proline (AcPro) at the amino terminus. P1-RiP is a reinitiated peptide lacking the amino-terminal AcPro. The amino acid sequence of “flag” is Asp–Tyr–Lys–Asp–Asp–Asp–Asp–Lys, which is translated from GAC-UAC-AAG-GAC-GAC-GAC-GAC-AAG. (*B*) MALDI-TOF mass spectrum of the translated peptides. The amino-terminal AcPro was introduced at the canonical initiation codon AUG using AcPro-tRNA^ini^_CAU_. Red and blue arrows indicate P1-FLP and P1-RiP, respectively. Calculated (calcd.) and observed (obsd.) *m/z* values of [M + H]^+^ are indicated. P1-FLP% was calculated using the regression line [1] shown in *C*. (*C*) Correlation between peak intensity ratio and concentration ratio of P1-FLP to P1-RiP. Mixtures of known concentration ratios of P1-FLP and P1-RiP were analyzed by MALDI-TOF MS to determine their peak intensity ratios. The equation of the regression line [1] and its correlation coefficient are shown in *bottom right*.

To induce the drop-off-reinitiation event in translation initiation, *N*-acetyl-L-proline (AcPro) was precharged on an initiator tRNA, tRNA^ini^, and introduced at the amino terminus of a model peptide P1 at the initiator codon of mRNA mR1 (Fig. 1A; Supplemental Fig. S1). For the preparation of AcPro-tRNA^ini^, Pro was charged on tRNA^ini^ by means of flexizyme (Goto et al. 2011) and its amino group was acetylated by acetic anhydride. To evaluate the specificity and efficiency of acetylation on Pro, a short tRNA analog, microhelix RNA (µhRNA), was subjected to a flexizyme reaction and/or acetic anhydride reaction, and then analyzed by MALDI-TOF mass spectrometry (MS) (Supplemental Fig. S2). Specific and complete acetylation was observed at the amino group of Pro charged on µhRNA. We then applied the same conditions to tRNA^ini^ for the preparation of AcPro-tRNA^ini^. We conducted translation of mR1 into P1 using an *Escherichia coli* reconstituted translation system, referred to as the flexible in vitro translation (FIT) system (Yamagishi et al. 2011), and observed both full-length P1 (P1-FLP) and reinitiated peptide lacking the amino-terminal AcPro (P1-RiP) by MALDI-TOF MS (Fig. 1B). This result indicates that the A-site Tyr-tRNA^Tyr^ moved to the P site by mistranslocation prior to completion of the peptidyl transfer reaction between the P-site AcPro-tRNA^ini^ and A-site Tyr-tRNA^Tyr^, followed by accommodation of the next Tyr-tRNA^Tyr^ onto the A site and eventually synthesis of P1-RiP. In order to determine the percentage of P1-FLP (FLP%), we analyzed the correlation between the peak intensity ratio (Y) and the concentration ratio (X) by measuring mixtures of synthetic P1-FLP and P1-RiP with known concentration ratios (Fig. 1C). We observed a linear relationship between log Y and log X with a regression line (1), whose correlation coefficient (R^2^) is 0.982.

(1) logY=1.027×logX+0.241.

**FIGURE 2.**
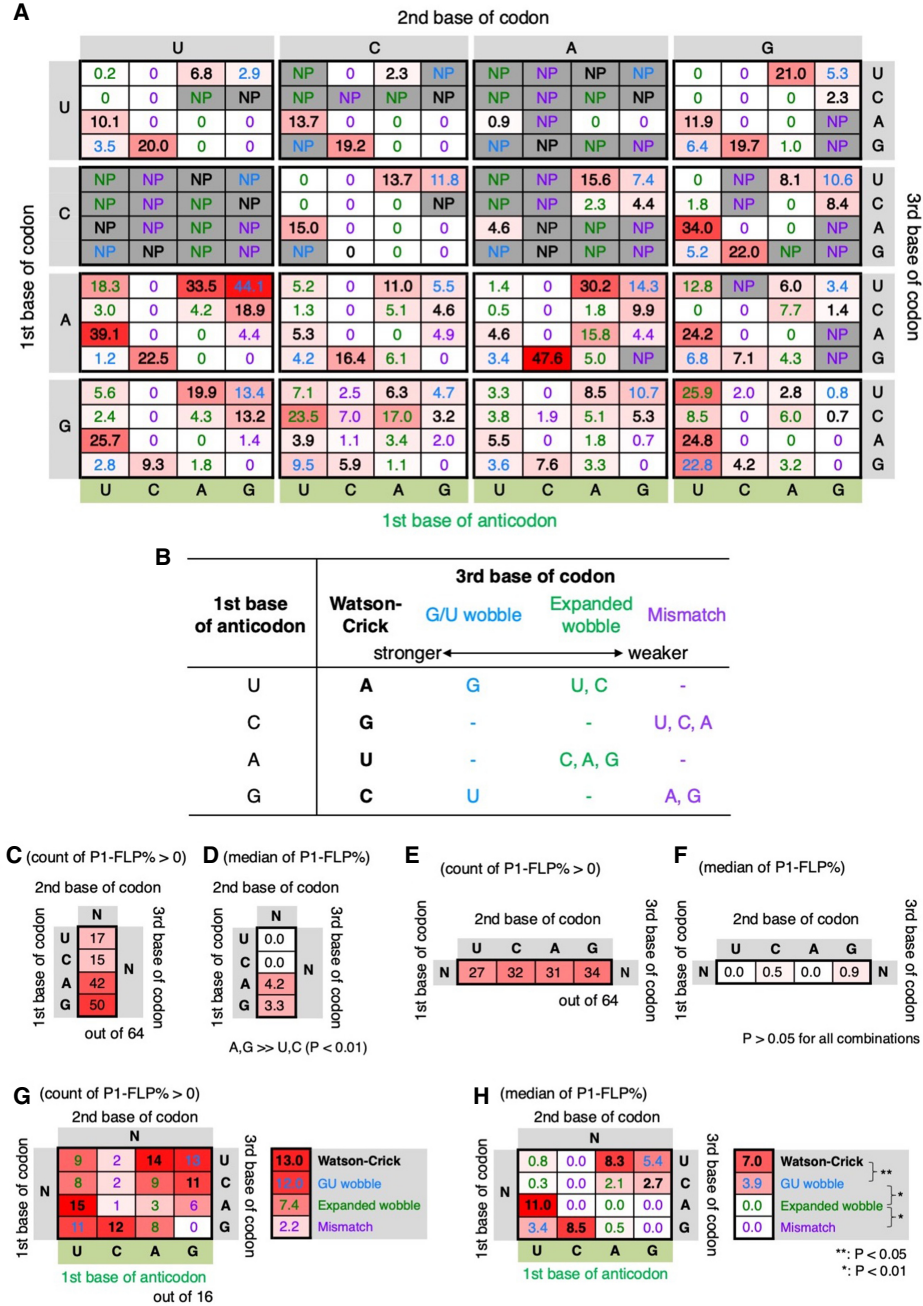
Analysis of P1-FLP% using 256 codon–anticodon combinations. (*A*) P1-FLP% of translation products using the 256 codon–anticodon combinations for incorporation of AcPro at the amino terminus. Average P1-FLP% values of three independent experiments are shown. Translation of the peptides was conducted in the FIT system containing 1.5 µM IF3, 0.1 µM EF-G, and 0.5 µM RRF. The first, second, and third base of the initiation codon are indicated at the *left*, *top*, and *right* side of the table, respectively. The first base of the anticodon is shown in the *bottom*. NP indicates no products, where neither full-length peptide nor truncated peptide were observed at all. Watson–Crick, G/U wobble, expanded wobble, and mismatch pairs are indicated by black, blue, green, and purple, respectively. (*B*) Classification of the type of base pairs. The base pairs between the first base of anticodon and the third base of codon were classified into four types: Watson–Crick, G/U wobble, expanded wobble, and mismatch pairs. (*C*,*E*,*G*) Number of codon–anticodon combinations that are able to yield full-length peptides. Impact of the first base (*C*), second base (*E*), and third base (*G*) of codon on the number of codon–anticodon combinations whose P1-FLP% > 0 are analyzed. For the third base of codon, a combination of the codon third base and the anticodon first base is also considered. The type of their interactions are classified into Watson–Crick, G/U wobble, expanded wobble, and mismatch base pairs, and their average values are indicated in the *right* panel (*G*). (*D*,*F*,*H*) Medians of P1-FLP% per type of the first base (*D*), second base (*F*), and third base (*H*). For the codon third base, medians of P1-FLP% per the base-pair type (Watson–Crick, G/U wobble, expanded wobble, and mismatch) are also indicated. *P*-values were estimated by the Mann–Whitney test.

Based on this regression line, P1-FLP% was calculated from the peak intensity ratio obtained by the MALDI-TOF MS of the translation products (Fig. 2A). For the canonical AUG initiator codon decoded by the CAU anticodon of tRNA^ini^, P1-FLP% was 22.5% (Fig. 1B; the peak intensity ratio obtained in this experiment was converted to P1-FLP% using the regression line [1]).

### Effect of codon/anticodon sequences on the frequency of amino-terminal drop-off-reinitiation

As the initiator codon, not only the canonical AUG but all of the 64 NNN codons were evaluated for AcPro incorporation at the amino terminus of P1-FLP. For decoding the NNN codons, the 64 tRNA^ini^s bearing NNN anticodons were tested. For the first and second base of the codon, only Watson–Crick base pairs were evaluated using complementary anticodon bases of tRNA. For the third base of the codon, 16 possible combinations of codon and anticodon bases were evaluated as summarized in Figure 2B, where the type of the base pairs were classified into four: Watson–Crick, G/U wobble, expanded wobble, and mismatched pairs (note that the strength of the base pair decreases in this order). Therefore, 256 codon–anticodon combinations were tested in total. When neither P1-FLP nor P1-RiP was observed, it is indicated by “NP” (no product) instead of the P1-FLP% value (Fig. 2A). Interestingly, codon AUG decoded by anticodon CAU is not the best codon–anticodon combination with the highest P1-FLP%; 12 codon–anticodon combinations showed higher P1-FLP% than AUG/CAU [Fig. 2A, CGA/UCG (34.0%), AUU/AAU (33.5%), AUU/GAU (44.1%), AUA/UAU (39.1%), AAU/AUU (30.2%), AAG/CUU (47.6%), AGA/UCU (24.2%), GUA/UAC (25.7%), GCC/UGC (23.5%), GGU/UCC (25.9%), GGA/UCC (24.8%), and GGG/UCC (22.8%)]. Nine of the 12 combinations have Watson–Crick base pairs at the codon third base, indicating that stronger base-pair interaction is preferred for the higher P1-FLP%; non-Watson–Crick base pairs exhibited generally lower P1-FLP% compared to Watson–Crick base pairs. To more precisely verify this, statistical analyses of the correlation between codon–anticodon combinations and their P1-FLP% were performed. In Figure 2C,E,G, the numbers of codon–anticodon combinations that were able to synthesize P1-FLP (P1-FLP% > 0) were counted per the type of first base (Fig. 2C), second base (Fig. 2E), and third base (Fig. 2G) of codons. Medians of P1-FLP% were also summarized per the type of the bases at each position (Fig. 2D,F,H). Figure 2C,D show that A and G at the first position of the codon exhibited significantly higher FLP% compared to U and C with the *P*-values <0.01. In contrast, the type of the second base of the codon was not significantly related to the P1-FLP% (Fig. 2E,F). For the codon third base, the type of base pairs was classified into four: Watson–Crick, G/U wobble, expanded wobble, and mismatch (Fig. 2G,H), where stronger base pairs showed significantly higher P1-FLP%. Their medians were 7.0, 3.9, 0.0, and 0.0, respectively (P < 0.05 for between Watson–Crick and G/U wobble, and P < 0.01 for other combinations). Among the Watson–Crick base pairs, the C/G pair showed significantly lower P1-FLP% than the others (Fig. 2H, 2.7 for C/G, whereas 8.3, 11.0, and 8.5 for U/A, A/U, and G/C, respectively).

**FIGURE 3.**
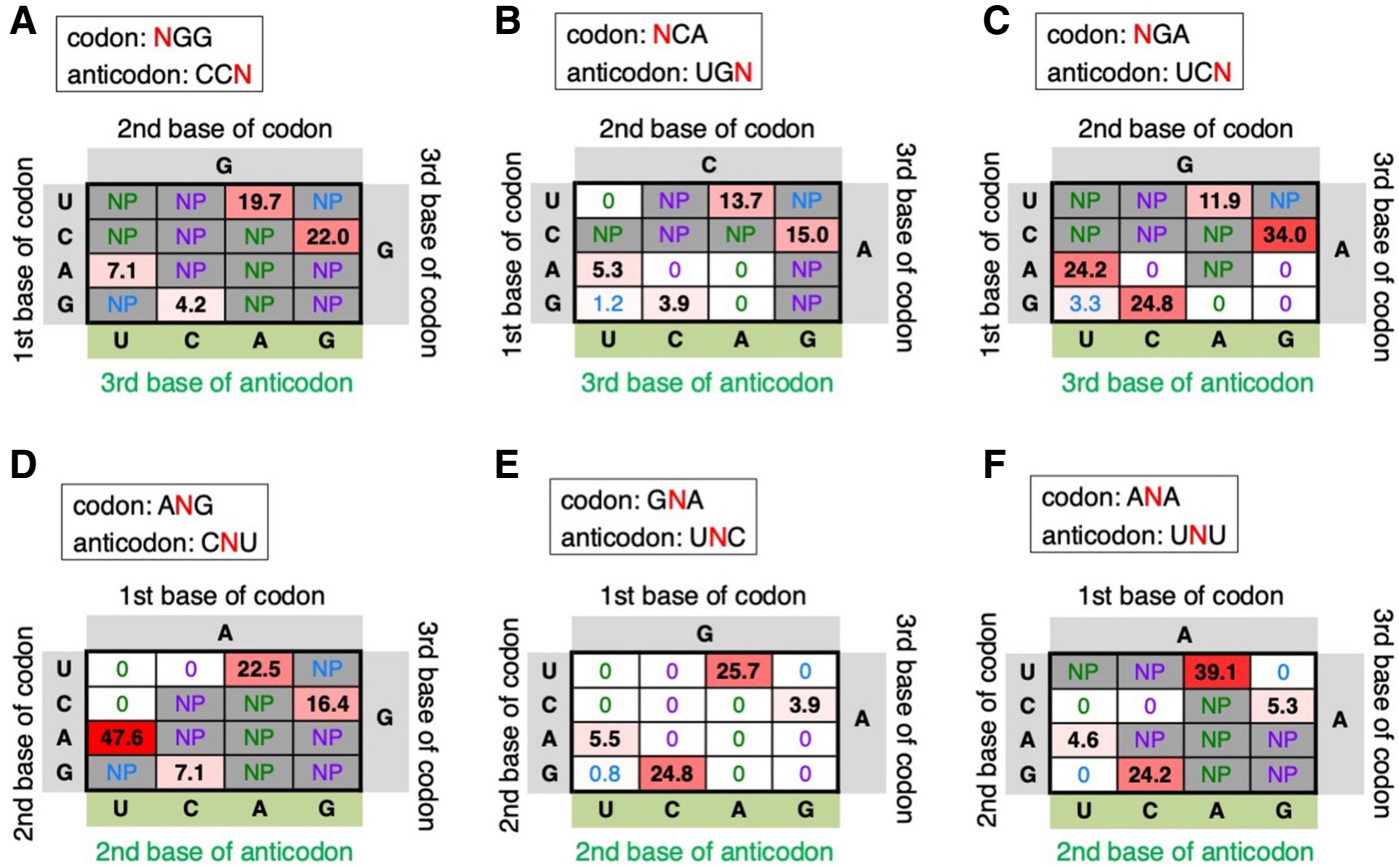
Evaluation of non-Watson–Crick base pairs at the first and second base of codon. (*A*−*C*) Evaluation of the first base of codon and the third base of anticodon. The first, second, and third bases of codons and the third bases of anticodons are indicated with their P1-FLP% values. Codon/anticodon for *A*, *B*, and *C* are NGG/CCN, NCA/UGN, and NGA/UCN, respectively. (*D*−*F*) Evaluation of the second base of codons and the second base of anticodons. Codon/anticodon for *D*, *E*, and *F* are ANG/CNU, GNA/UNC, and ANA/UNU, respectively.

Next, non-Watson–Crick base pairs at the first and second positions of the codon were also evaluated for some representative sequences (Fig. 3). Sixteen each codon–anticodon combinations for NGG/CCN (Fig. 3A), NCA/UGN (Fig. 3B), NGA/UCN (Fig. 3C), ANG/CNU (Fig. 3D), GNA/UNC (Fig. 3E), and ANA/UNU (Fig. 3F) were evaluated. In these combinations, most of non-Watson–Crick base pairs resulted in no P1-FLP synthesis (0% or NP) except for some G/U wobble base pairs with very low P1-FLP% (1.2%, 3.3%, and 0.8% for GCA/UGU, GGA/UCU, and GGA/UUC, respectively), indicating that stable base-pair formation, that is, Watson–Crick base pair, is required at the first and second positions of the codon for P1-FLP synthesis. In contrast, as shown above, many of the non-Watson–Crick base pairs are tolerated at the codon third base for P1-FLP synthesis, albeit their relatively low P1-FLP% in general, indicating that those base pairs were tolerated by IF3 for placing initiator tRNA at the active position.

### Regulation of the drop-off-reinitiation frequency by IF3

**FIGURE 4.**
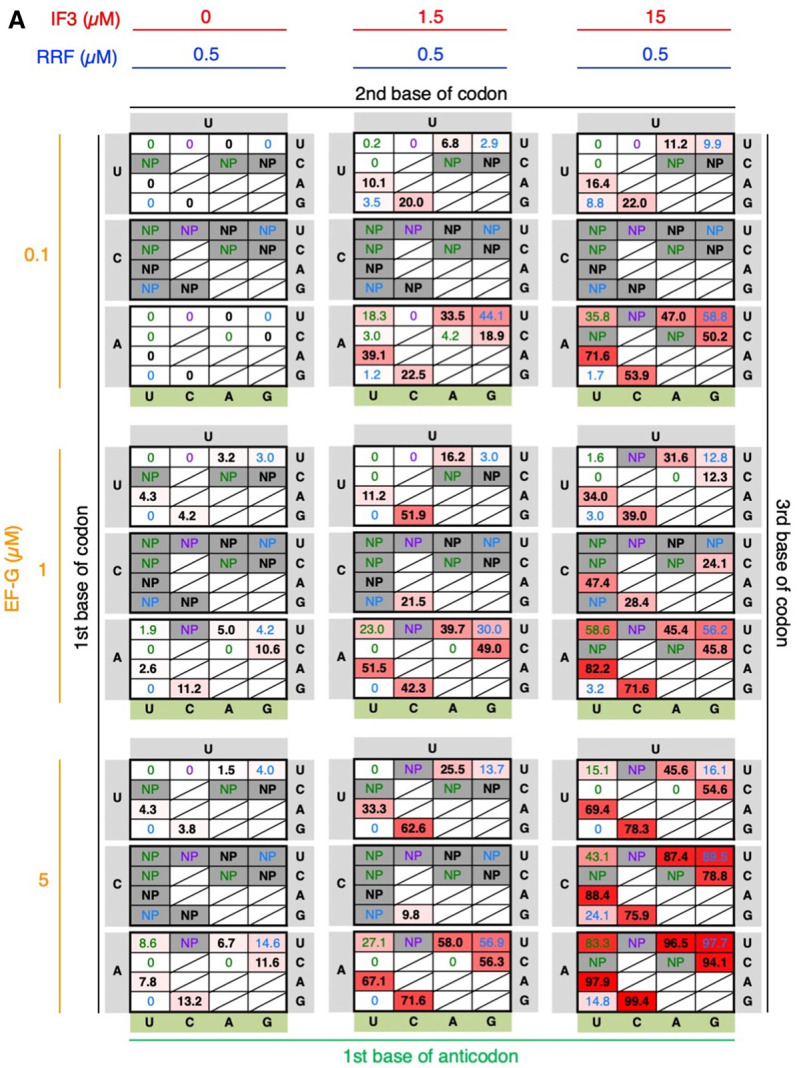

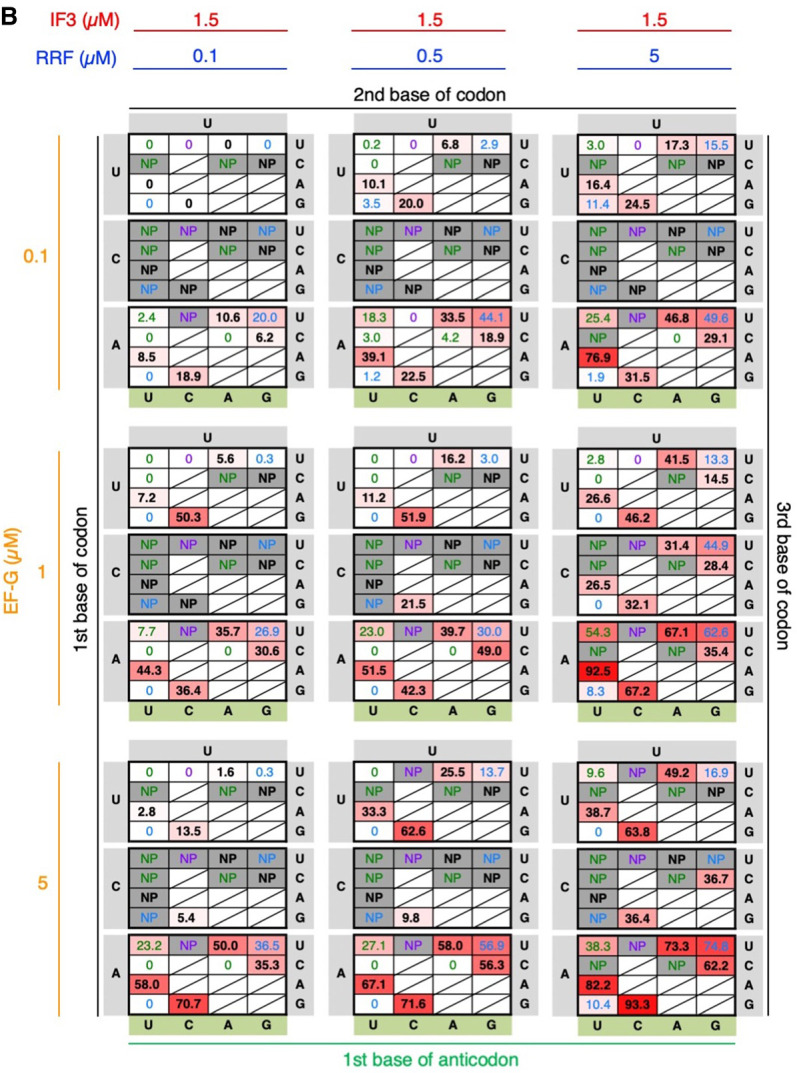
Titration of IF3, EF-G, and RRF in incorporation of AcPro at the amino terminus of P1. (*A*) Titration of IF3 and EF-G with 0.5 µM RRF. (*B*) Titration of RRF and EF-G with 1.5 µM IF3. P1-FLP% for incorporation of AcPro at HUN codons using NAD anticodon was evaluated with titration of IF3, EF-G, and RRF concentrations. Average P1-FLP% values of three independent experiments are shown. NP stands for no products, where neither full-length peptide nor truncated peptide were observed. Boxes with *diagonal* lines indicate that P1-FLP% was not examined. Watson–Crick, G/U wobble, expanded wobble, and mismatch base pairs are indicated by black, blue, green, and purple, respectively. Note that the P1-FLP% values for 1.5 µM IF3/0.5 µM RRF are identical in parts *A* and *B*.

Here, we assumed that if IF3 fails to recognize the codon–anticodon interaction to place AcPro-tRNA^ini^ at the active position, then P1-FLP% should decrease due to the increased frequency of the amino-terminal drop-off-reinitiation. In order to verify this, IF3 concentration was titrated for P1-FLP/P1-RiP synthesis (Fig. 4A, IF3 = 0, 1.5. and 15 µM, EF-G = 0.1 µM, RRF = 0.5 µM). Note that the standard concentrations of IF3, EF-G, and RRF in the FIT system are 1.5, 0.1, and 0.5 µM, respectively. In this analysis, we focused on HUN codons (H = U, C, or A) decoded by NAD anticodons (D = U, A, or G), where H and D form Watson–Crick base pairs. The following combinations were omitted: HUC/CAD, HUA/VAD, and HUG/RAD (indicated by slash in Fig. 4). In the absence of IF3 (=0 µM), P1-FLP could not be synthesized at all for all the codon–anticodon combinations (0% or NP). In contrast, at the higher IF3 concentration (15 µM), P1-FLP% was improved compared to the standard concentration (1.5 µM) for the stronger base pairs, like Watson–Crick (UUU/AAA, UUA/UAA, UUG/CAA, AUU/AAU, AUC/GAU, AUA/UAU, and AUG/CAU) and G/U wobble base pairs (UUU/GAA, UUG/UAA, AUU/GAU, and AUG/UAU), whereas P1-FLP% decreased for the weaker base pair, such as UUU/UAA, AUC/UAU, and AUC/AAU. These results indicate that IF3 preferentially recognizes stronger base pairs, that is, Watson–Crick and G/U wobble base pairs, regardless of their sequences, to place AcPro-tRNA^ini^ at the active position. For the canonical AUG/CAU pair, P1-FLP% increased from 22.5% to 53.9%. On the other hand, weaker base pairs cannot be well recognized by IF3 and therefore fail to be activated, resulting in low P1-FLP%. The only exception was AUU/UAU (expanded wobble, 35.8%).

### Involvement of EF-G and RRF in regulation of the amino-terminal drop-off-reinitiation

The drop-off-reinitiation event in elongation is induced by EF-G, because EF-G mediates the mistranslocation of P-site peptidyl-tRNA and A-site aminoacyl-tRNA into the E site and P site, respectively, prior to completion of peptidyl transfer (Tajima et al. 2022). Thus, here we have assumed that the amino-terminal drop-off-reinitiation could also be accelerated at higher concentrations of EF-G due to mistranslocation of the P-site AcPro-tRNA^ini^ and A-site Tyr-tRNA^Tyr^. To verify this assumption, we conducted a titration experiment of EF-G in P1-FLP/P1-RiP synthesis (Fig. 4A, EF-G = 0.1, 1, and 5 µM, IF3 = 1.5 µM, RRF = 0.5 µM). Although we expected P1-FLP% would generally decrease at higher EF-G concentrations, to our surprise P1-FLP% significantly increased at higher EF-G concentrations for the stronger base pairs, for example, Watson–Crick (UUU/AAA, UUA/UAA, UUG/CAA, AUU/AAU, AUC/GAU, AUA/UAU, and AUG/CAU) and G/U wobble base pairs (UUU/GAA and AUU/GAU) (Fig. 4A, EF-G = 1 or 5 µM, IF3 = 1.5 µM, RRF = 0.5 µM). In contrast, for the weaker base pairs, i.e., expanded wobble and mismatch (UUU/UAA, AUC/UAU, and AUC/AAU), P1-FLP% decreased. These results were similar to those of IF3 titration, showing that EF-G is also involved in increasing the ratio of P1-FLP, depending on the strength of codon–anticodon interaction. Then, we also conducted titration of both IF3 and EF-G at the same time (Fig. 4A, IF3 = 0, 1.5, and 15 µM, EF-G = 0.1, 1, and 5 µM, RRF = 0.5 µM). Although P1-FLP synthesis was quite inefficient for CUN codons at lower IF3 and EF-G concentrations (only NP with 1.5 µM IF3 and 0.1 µM EF-G), we observed a drastic increase of P1-FLP% for CUN codons at higher IF3 and EF-G concentrations like 15 µM IF3 and 5 µM EF-G (88.4% for CUA/UAG, for example). As for the canonical AUG/CAU, P1-FLP% remarkably reached 99.4% with 15 µM IF3 and 5 µM EF-G, indicating the synergetic effect of IF3 and EF-G.

For the role of EF-G in alleviation of the amino-terminal drop-off-reinitiation, we hypothesized that aberrant 70S IC with inactive AcPro-tRNA^ini^ is disassembled and recycled by EF-G. If that is the case, the inactive AcPro-tRNA^ini^ is not utilized for translation initiation and thereby the drop-off-reinitiation event can be circumvented. As it is known that EF-G and RRF cooperatively disassemble post-termination ribosomal complexes (Hirashima and Kaji 1972; Zavialov et al. 2005), aberrant 70S IC with inactive AcPro-tRNA^ini^ could also be disassembled by a similar mechanism. To verify this hypothesis, we have tested the possibility that RRF is also involved in alleviation of the drop-off-reinitiation along with EF-G. First, synthesis of P1 was conducted with titration of RRF (Fig. 4B, RRF = 0.1, 0.5, and 5 µM, EF-G = 0.1 µM, and IF3 = 1.5 µM). By increasing the RRF concentration from 0.1 to 0.5 and then to 5 µM, P1-FLP% significantly increased for Watson–Crick (UUU/AAA, UUA/UAA, UUG/CAA, AUU/AAU, AUC/GAU, AUA/UAU, and AUG/CAU) and G/U wobble base pairs (UUU/GAA, UUG/UAA, and AUU/GAU). We also performed the titration of both EF-G and RRF at the same time (Fig. 4B, EF-G = 0.1, 1, and 5 µM, RRF = 0.1, 0.5, and 5 µM, and IF3 = 1.5 µM). By increasing the concentrations of EF-G and RRF up to 5 µM each, P1-FLP% for the stronger base pairs (Watson–Crick and G/U wobble) further increased. For instance, P1-FLP% of the canonical AUG/CAU reached 93.3% even though the IF3 concentration is not high (1.5 µM). These results show both EF-G and RRF are involved in alleviation of the amino-terminal drop-off-reinitiation by disassembling 70S IC with inactive AcPro-tRNA^ini^ whose codon–anticodon interaction is weak.

### Drop-off-reinitiation induced by N-formylmethionine incorporation

**FIGURE 5.**
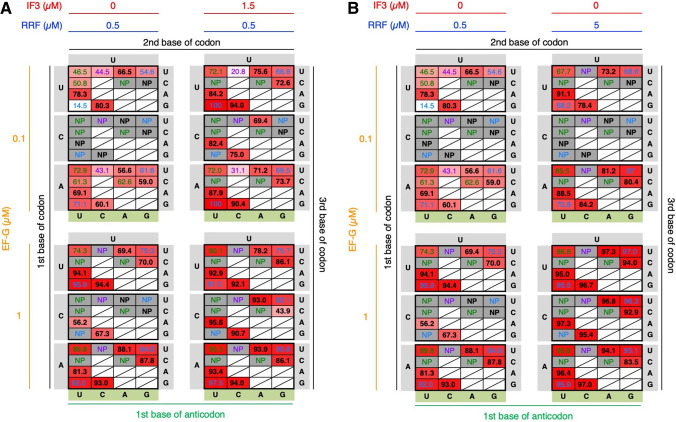
Titration of IF3, EF-G, and RRF in incorporation of fMet at the amino terminus of P1. (*A*) Titration of IF3 and EF-G with 0.5 µM RRF. (*B*) Titration of RRF and EF-G with 0 µM IF3. P1-FLPfM% for incorporation of fMet at HUN codons using NAD anticodon was evaluated with titration of IF3, EF-G, and RRF concentrations. Average P1-FLPfM% values of three independent experiments are shown. NP stands for no products, where neither full-length peptide nor truncated peptide were observed. Boxes with *diagonal* lines indicate that P1-FLP% was not examined. Watson–Crick, G/U wobble, expanded wobble, and mismatch base pairs are indicated by black, blue, green, and purple, respectively. Note that the P1-FLPfM% values for 0 µM IF3/0.5 µM RRF are identical in parts *A* and *B*.

We next observed drop-off-reinitiation by introducing the canonical initiator substrate, N-formylmethionine (fMet), at the amino terminus of P1 peptide (Fig. 5; Supplemental Fig. S3). Since the reactivity of fMet as a peptidyl donor should be higher than that of AcPro, IF3 was omitted from the translation system to induce drop-off-reinitiation, whereas the concentrations of EF-G and RRF remained 0.1 and 0.5 µM, respectively. Consequently, both full-length peptide (P1-FLPfM) and reinitiated peptide (P1-RiP) were observed for the canonical AUG/CAU by MALDI-TOF MS (Supplemental Fig. S3B). To determine the percentage of P1-FLPfM (P1-FLPfM%), we analyzed the peak intensity ratios (Y) of mixtures of synthetic P1-FLPfM and P1-RiP with known concentration ratios (X), and thereby a regression line (2) was obtained (Supplemental Fig. S3C).

(2) logY=1.050×logX+0.201.

Using Equation 2, P1-FLPfM% was estimated to be 60.1% for AUG/CAU. Since the P1-FLP% for AcPro incorporation under the same translation conditions (IF3 = 0 µM) was 0% (Fig. 4), it was confirmed that fMet incorporation is much more efficient than AcPro incorporation. This result also indicates that IF3 is not indispensable for fMet incorporation at the amino terminus; even if fMet-tRNA^ini^ could not be placed at the optimal position in the absence of IF3, peptidyl transfer between fMet-tRNA^ini^ and Tyr-tRNA^Tyr^ still occurs owing to the higher reactivity of fMet.

To exclude the possibility that formylation efficiency decreases due to the introduction of G1 in place of the canonical C1 at the 5′ end of tRNA^ini^ (Supplemental Fig. S1), P1-FLPfM% for AUG/CAU combination was compared between G1 and C1 variants (Supplemental Fig. S3D). Consequently, the G1 variant showed comparable P1-FLPfM% to that of C1 (90.4% and 84.1% for G1 and C1, respectively), indicating that *E. coli* methionyl-tRNA formyltransferase (MTF) is able to catalyze formylation on both G1 and C1 to a similar level.

To analyze the relationship between the drop-off-reinitiation frequency and the codon/anticodon combinations, not only AUG/CAU but 30 codon/anticodon combinations were evaluated for fMet incorporation at the amino terminus of P1-FLPfM (Fig. 5, HUN codons decoded by NAD anticodons except for HUC/CAD, HUA/VAD, and HUG/RAD, where H and D form Watson–Crick base pairs). In translation with 0 µM IF3, 0.1 µM EF-G, and 0.5 µM RRF, not only Watson–Crick base pairs but also most of G/U wobble, expanded wobble, and mismatch base pairs exhibited moderate P1-FLPfM% (mainly 40%−80%), except for CUN codons and UUC/RAA that resulted in NP. On the other hand, by increasing the IF3 concentration up to 1.5 µM, P1-FLPfM% of Watson–Crick and G/U wobble base pairs increased up to 70%−100%, whereas those of expanded wobble and mismatch pairs significantly decreased to 20.8% (UUU/CAA), 31.1% (AUU/CAU), and NP (UUC/UAA, AUC/UAU, and AUC/AAU) except for UUU/UAA and AUU/UAU (Fig. 5A). Higher concentrations of EF-G (1 µM) and RRF (5 µM) also showed similar tendencies: increase of P1-FLPfM% for Watson–Crick and G/U wobble base pairs and decrease of P1-FLPfM% for expanded wobble and mismatch pairs except for WUU/UAW (Fig. 5B). These results show that the three translation factors, IF3, EF-G, and RRF, are involved in alleviation of the amino-terminal drop-off-reinitiation as is the case with AcPro incorporation. A combination of U at the codon third base and U at the anticodon first base seems to be relatively well activated by IF3, EF-G and RRF among the expanded wobble base pairs; again, the same tendency was observed for AcPro incorporation.

Since GUG and UUG codons are occasionally used as initiation codons in *E. coli* (Villegas and Kropinski 2008; Hecht et al. 2017), decoding of these codons by CAU anticodon was evaluated for P1-FLPfM synthesis (Supplemental Fig. S3D, 73.8%, 55.8%, and 90.4% P1-FLPfM for GUG, UUG, and AUG, respectively, in the presence of 1.5 µM IF3, 0.1 µM EF-G, and 0.5 µM RRF). Drop-off-reinitiation was induced more frequently with a mismatch pair at the first base of the codon (UUG/CAU) and a G/U wobble pair (GUG/CAU) in this order, while less frequently with a Watson–Crick pair (AUG/CAU).

### Evaluation of peptide expression level with titration of IF3, EF-G, and RRF

**FIGURE 6.**
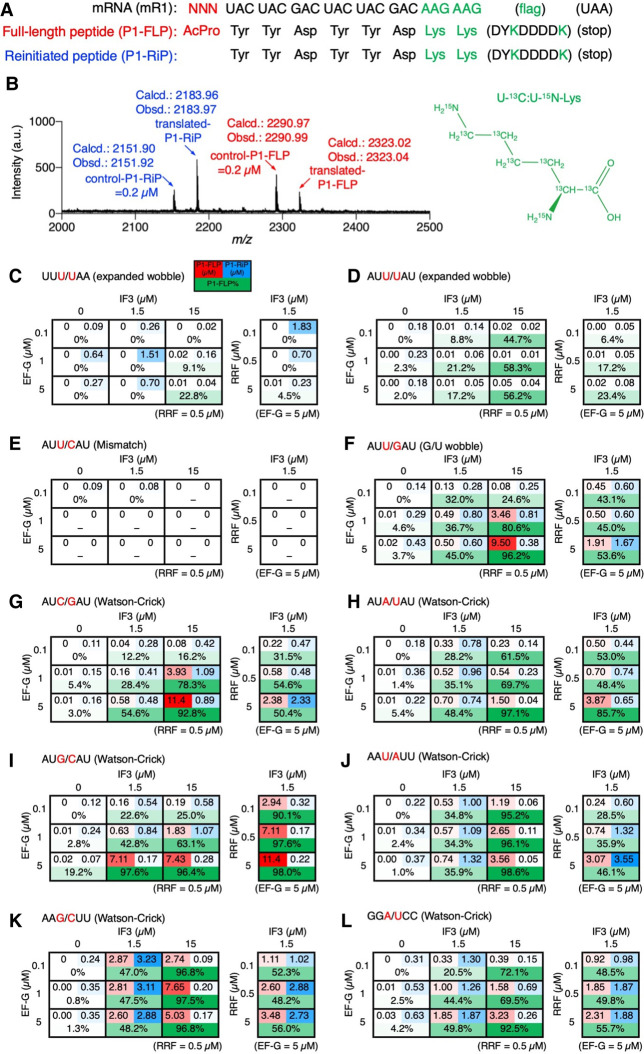
Quantification of expression levels of P1-FLP and P1-RiP. (*A*) Sequences of mRNA, mR1, and the corresponding peptide sequences P1-FLP and P1-RiP, where AAG codons were used for incorporation of isotope-labeled Lys (U-^13^C:U-^15^N-Lys) and indicated by green. (*B*) MALDI-TOF MS of translated peptides P1-FLP and P1-RiP translated. Translation was carried out in the presence of U-^13^C:U-^15^N-Lys (monoisotopic mass: 154.12) instead of unlabeled Lys (monoisotopic mass: 146.11). An amount of 0.2 µM each of synthetic internal control peptides, control-P1-FLP and control-P1-RiP, bearing unlabeled Lys were added to the translation system. Calculated (Calcd.) and observed (Obsd.) [M + H]^+^ values of translated-P1-FLP, translated-P1-RiP, control-P1-FLP, and control-P1-RiP are indicated. (*C*−*L*) Expression levels of P1-FLP and P1-RiP, and their ratio (P1-FLP%) for 10 representative codon–anticodon combinations. P1-FLP (µM), P1-RiP (µM), and P1-FLP% are shown at the *top left*, *top right*, and *bottom* in each box, respectively, and their intensities are indicated by red, blue, and green heatmaps, respectively. Average values of three independent experiments are shown. Concentrations of IF3, EF-G, and RRF used for translation are also indicated.

In the above experiments, only the ratios of full-length peptides were evaluated; however, their expression levels could also be improved by alleviating the drop-off-reinitiation by elevating the concentrations of IF3, EF-G, and RRF. To measure the expression level of peptides, translation was carried out in the presence of U-^13^C:U-^15^N-Lys (M.W. 154.12) instead of unlabeled Lys (M.W. 146.11), so that the translated peptides have four U-^13^C:U-^15^N-Lys residues (Fig. 6A). In addition, 0.2 µM each of synthetic internal control peptides, control-P1-FLP and control-P1-RiP, bearing unlabeled Lys, were also added to the translation system. [M + H]^+^ values of translated-P1-FLP and translated-P1-RiP are 2323.02 and 2183.96, respectively, and those of control-P1-FLP and control-P1-RiP are 2290.97 and 2151.90, respectively. Since translated-P1-FLP and control-P1-FLP have the identical amino acid sequence, except for the isotope labeling, their ionization efficiency in MALDI-TOF MS should be nearly equal to each other. Therefore, we can determine the concentration of translated-P1-FLP by the peak intensity ratio of translated-P1-FLP to control-P1-FLP. The concentration of translated-P1-RiP can also be determined the same way. For instance, the expression levels of P1-FLP and P1-RiP for the canonical AUG/CAU were determined to be 0.16 and 0.54 µM, respectively, in the presence of 1.5 µM IF3, 0.1 µM EF-G, and 0.5 µM RRF (Fig. 6B).

By using this method, the expression levels of P1-FLP and P1-RiP were determined for ten representative codon–anticodon combinations with titration of IF3, EF-G, and RRF (Fig. 6C−L, UUU/UAA, AUU/UAU, AUU/CAU, AUU/GAU, AUC/GAU, AUA/UAU, AUG/CAU, AAU/AUU, AAG/CUU, and GGA/UCC). In the case of relatively stronger base pairs (Watson–Crick and G/U wobble) at the codon third base, not only P1-FLP% but their expression level was generally improved up to 3−11 µM by increasing any of IF3, EF-G, and RRF concentrations. In contrast, in the case of weaker base pairs (expanded wobble and mismatch), the expression levels of P1-FLP were extremely low in general (at most 0.05 µM), even if the P1-FLP% was relatively high (Fig. 6D, 56.2% for AUU/UAU with 15 µM IF3, 5 µM EF-G, and 0.5 µM RRF). This tendency was observed regardless of codon/anticodon sequences, indicating that IF3, EF-G, and RRF monitor the strength of codon–anticodon interactions rather than their sequences.

## DISCUSSION

In this study, we revealed that drop-off-reinitiation occurs at the amino terminus of nascent peptides using AcPro and the canonical fMet as the amino-terminal substrates. We previously reported that peptidyl-tRNAs with shorter nascent peptides are more prone to cause drop-off-reinitiation in elongation likely because their weaker interaction with the ribosomal tunnel leads to higher drop-off frequency. Since the initiator aminoacyl-tRNAs used in this study, AcPro-tRNA^ini^ and fMet-tRNA^ini^, comprise only one amino acid, their interaction with the ribosomal tunnel should be weaker than that of longer peptidyl-tRNAs utilized for elongation events. Therefore, drop-off of AcPro-tRNA^ini^ or fMet-tRNA^ini^ is more likely to occur and induce the amino-terminal drop-off-reinitiation. Moreover, much lower reactivity of AcPro as the P-site substrate compared to fMet makes drop-off-reinitiation more frequent. Therefore, we observed generally lower P1-FLP% when introducing AcPro at the amino terminus than P1-FLPfM% for introduction of fMet. Indeed, to induce frequent drop-off-reinitiation with fMet, we needed to conduct translation in the absence of IF3 with low EF-G and RRF concentrations. It should be noted that the use of in vitro transcribed tRNA^ini^ lacking nucleotide modification might cause inefficient translation. Thus, there is a possibility that the P1-FLPfM% obtained in our study could be underestimated compared to those in the native translation system.

If IF3 fails to place fMet-tRNA^ini^ at an active position, the inactive fMet-tRNA^ini^ would become more prone to cause drop-off-reinitiation due to its instability at the P site. However, previous biochemical/structural studies have mainly focused on the canonical AUG/CAU codon–anticodon pair, and only limited experimental evidence has been accumulated for other codon–anticodon combinations. For instance, Antoun et al. reported that IF3 destabilizes the binding of fMet-tRNA to the 30S subunit for AUG/CAU combination; however, it is unclear whether this applies to the other codon–anticodon combinations. Therefore, here we have performed for the first time a comprehensive study of 328 codon–anticodon combinations to examine the relationship between P1-FLP% and the contribution of IF3. As a result, we were able to reveal the following rules for the preference of codon–anticodon combinations by IF3: (i) Watson–Crick base-pair formation is required at the first and second base of the codon. (ii) For the third base of the codon, not only Watson–Crick but other types of base pairs are tolerated with a correlation between the strength of base pairs and tolerance. For the G/U wobble, the combination of codon U and anticodon G is more tolerated than that of codon G and anticodon U. Expanded wobble and mismatch base pairs are by far less tolerated.

**FIGURE 7.**
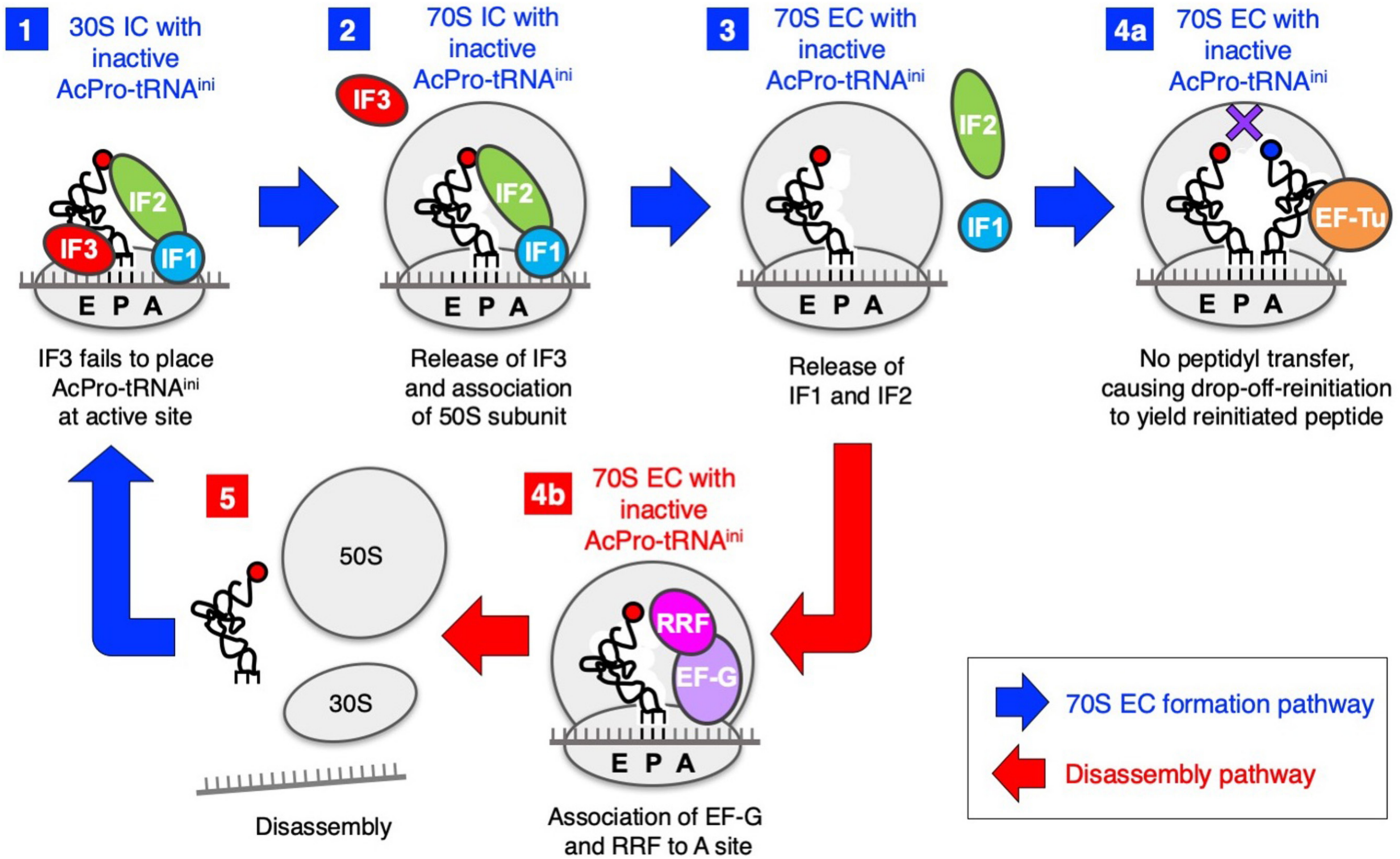
Overview of the roles of IF3, EF-G, and RRF in the amino-terminal drop-off-reinitiation. Formation of 70S EC with inactive AcPro-tRNA^ini^ and its disassembly by EF-G and RRF are illustrated. (1) Formation of 30S IC with inactive AcPro-tRNA^ini^, where IF3 fails to place AcPro-tRNA^ini^ at active site. (2) Dissociation of IF3 from the 30S IC and association of 50S ribosomal subunit to form 70S IC with inactive AcPro-tRNA^ini^. (3) Dissociation of IF1 and IF2 to form 70S EC with inactive AcPro-tRNA^ini^. (4a) Accommodation of A-site aminoacyl-tRNA mediated by EF-Tu, followed by drop-off-reinitiation to give a reinitiated peptide; (4b) association of EF-G and RRF to the ribosomal A site. (5) Disassembly of 70S EC.

EF-G and RRF are also involved in alleviation of the amino-terminal drop-off-reinitiation. This is not the case with the drop-off-reinitiation in the elongation event, which is enhanced by EF-G and not affected by RRF (Tajima et al. 2022). Thus, drop-off-reinitiation in initiation and elongation are differently regulated. It has been reported that EF-G and RRF cooperatively disassemble post-termination ribosomal complexes. Therefore, we assumed that EF-G and RRF are involved in disassembly of aberrant 70S EC with inactive fMet-tRNA^ini^ or AcPro-tRNA^ini^ to recycle the ribosome for alleviation of drop-off-reinitiation (Fig. 7, step 3 to 4b, and then to 5). Given that codon–anticodon dependence and EF-G/RRF concentration dependence are observed for alleviation of drop-off-reinitiation, 70S EC with less stable fMet-tRNA^ini^/AcPro-tRNA^ini^ should be more rapidly disassembled in an EF-G/RRF concentration-dependent manner.

Under our standard translation conditions, i.e., 1.5 µM IF3, 0.1 µM EF-G, and 0.5 µM RRF, P1-FLP% for the canonical AUG/CAU was only 22.5% and the expression level of P1-FLP was 0.16 µM, whereas with higher concentrations of these translation factors (15, 5, and 5 µM, respectively), P1-FLP% and the expression level were improved up to 96.4%−98.0% and 7.1−11.4 µM, respectively (Figs. 2, 6I). These results indicate the possibility that ribosomal incorporation of inefficient initiator building blocks such as AcPro can be drastically improved by optimizing IF3, EF-G, and RRF concentrations, which is beneficial for translation of exotic peptides with noncanonical initiator building blocks.

## MATERIALS AND METHODS

### Preparation of aminoacyl-tRNA

tRNA^ini^ and flexizyme (dFx) were prepared by PCR and in vitro transcription. Template DNAs used for transcription were prepared by extension of forward and reverse extension primer pairs, followed by PCR using forward and reverse PCR primers (see Supplemental Table S1 for the sequences of primers). Transcription reaction was carried out at 37°C for 16 h in 250 µL (tRNA^ini^) or 2 mL (dFx) reaction mixtures containing 40 mM Tris-HCl (pH 8.0), 22.5 mM MgCl_2_, 1 mM DTT, 1 mM spermidine, 0.01% Triton X-100, 5 mM NTP mix, 0.04 U/µL RNasin RNase inhibitor (Promega), and 0.12 µM T7 RNA polymerase. For the transcription of tRNA^ini^, the concentration of NTP mix was modified to 3.75 mM, and 5 mM guanosine monophosphate was added. The resulting RNAs were treated with RQ1 DNase (Promega) for 30 min at 37°C and purified on 8% (tRNA^ini^) or 12% (dFx) polyacrylamide gels containing 6 M urea.

Activated amino acids [l-proline-3,5-dinitrobenzyl ester (l-Pro-DBE) and l-methionine-3,5-dinitrobenzyl ester (l-Met-DBE)] were synthesized by previously reported methods (Saito et al. 2001; Murakami et al. 2006). Aminoacylation was carried out at 0°C for 2 h in a reaction mixture containing 50 mM HEPES-KOH (pH 7.5), 600 mM MgCl_2_, 20% DMSO, 25 μM dFx, 25 μM tRNA^ini^ or µhRNA, and 5 mM activated amino acids. The aminoacyl-tRNAs were recovered by ethanol precipitation, washed twice with 70% ethanol containing 0.1 M sodium acetate (pH 5.2), and dissolved in 1 mM sodium acetate (pH 5.2). For preparation of AcPro-tRNA^ini^ and AcPro-µhRNA, 250 pmol Pro-tRNA^ini^ or Pro-µhRNA was dissolved in 60 µL 0.3 M sodium acetate/0.5 M acetic anhydride solution (pH 5.2), incubated for 30 min at 25°C, and then recovered by ethanol precipitation. The pellet was washed twice with 70% ethanol containing 0.1 M sodium acetate (pH 5.2), and dissolved in 1 mM sodium acetate (pH 5.2).

### In vitro translation of model peptides

In vitro translation of peptides P1-FLP or P1-FLPfM and P1-RiP was carried out at 37°C for 30 min in the FIT system containing the following components: 50 mM HEPES-KOH (pH 7.6), 100 mM potassium acetate, 12.6 mM magnesium acetate, 2 mM ATP, 2 mM GTP, 1 mM CTP, 1 mM UTP, 20 mM creatine phosphate, 2 mM spermidine, 1 mM DTT, 1.5 mg/mL *E. coli* total tRNA, 1.2 µM *E. coli* ribosome, 0.6 µM MTF, 2.7 µM IF1, 3 µM IF2, 1.5 µM IF3, 0.1 µM EF-G, 20 µM EF-Tu/Ts, 0.25 µM RF2, 0.17 µM RF3, 0.5 µM RRF, 4 µg/mL creatine kinase, 3 µg/mL myokinase, 0.1 µM inorganic pyrophosphatase, 0.1 µM nucleotide diphosphate kinase, 0.1 µM T7 RNA polymerase, 0.13 µM AspRS, 0.11 µM LysRS, 0.02 µM TyrRS, 0.5 mM Asp, 0.5 mM Lys, 0.5 mM Tyr, 20 µM AcPro-tRNA^ini^ or Met-tRNA^ini^, and 0.5 µM DNA template. The DNA template used for translation was prepared by extension of forward and reverse extension primer pairs, followed by PCR using forward and reverse PCR primer pairs (see Supplemental Table S1 for the sequences of primers). For incorporation of fMet at the amino terminus, 0.1 mM 10-formyl-5,6,7,8-tetrahydrofolic acid was added to the above reaction mixture. Note that *N*-formylation of Met-tRNA^ini^ was performed in parallel in the translation system using 10-formyl-5,6,7,8-tetrahydrofolic acid and methionyl-tRNA formyltransferase.

### MALDI-TOF mass spectroscopic analysis of model peptide and microhelix RNA

The above translation reaction mixture was desalted with SPE C-tip (Nikkyo Technos) and cocrystallized with α-cyano-4-hydroxycinnamic acid. MALDI-TOF MS analyses were performed using UltrafleXtreme (Bruker Daltonics) in a reflector-positive mode. A peptide calibration standard II (Bruker Daltonics) was used for external mass calibration. For analysis of µhRNA, 3-hydroxypicolinic acid was used for cocrystallization and MALDI-TOF MS was conducted in a linear-positive mode.

## SUPPLEMENTAL MATERIAL

Supplemental material is available for this article.

## Supplementary Material

Supplemental Material
