# Supplementary material for: Drop-off-reinitiation at the amino termini of nascent peptides and its regulation by IF3, EF-G, and RRF
Source: RNA. 2023 May;29(5):663–74. doi: 10.1261/rna.079447.122 (PMC10158994; doi:10.1261/rna.079447.122)
Supplement: Supplemental Material [file supp_29_5_663__DC1.html]

Drop-off-reinitiation at the amino termini of nascent peptides and its regulation by IF3, EF-G, and RRF — Supplemental Material 

# Drop-off-reinitiation at the amino termini of nascent peptides and its regulation by IF3, EF-G, and RRF

## Supplemental Material

- Supplemental\_Figure\_S1.pdf
- Supplemental\_Figure\_S2.pdf
- Supplemental\_Figure\_S3.pdf
- Supplemental\_Table\_S1.xls
