# Supplementary material for: Drop-off-reinitiation at the amino termini of nascent peptides and its regulation by IF3, EF-G, and RRF
Source: RNA. 2023 May;29(5):663–74. doi: 10.1261/rna.079447.122 (PMC10158994; doi:10.1261/rna.079447.122)
Supplement: Supplemental Material [file supp_079447.122_Supplemental_Figure_S2.pdf]

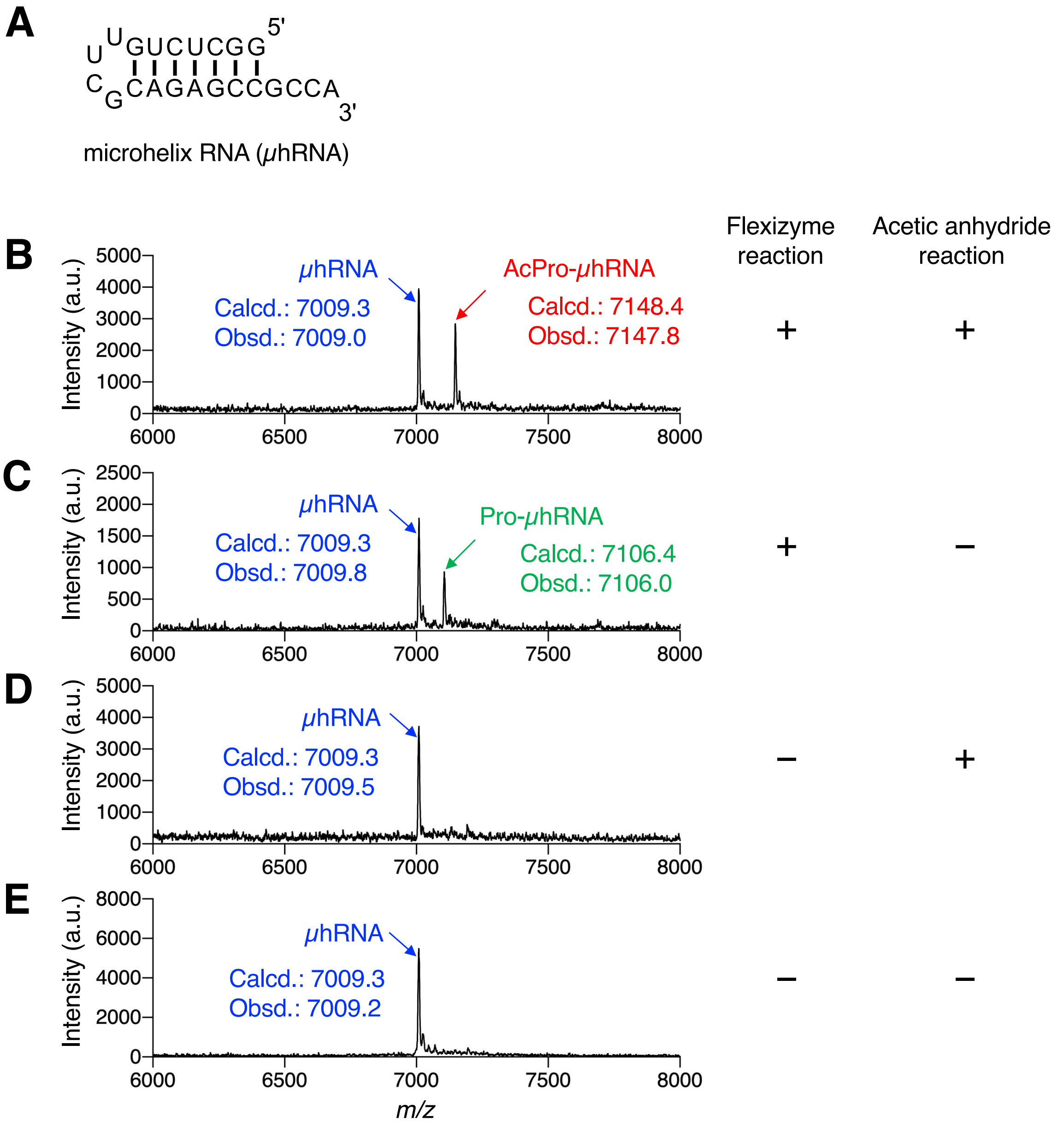

SUPPLEMENTAL FIGURE S2. Acetylation of Pro charged on microhelix RNA using acetic anhydride. (A) Secondary structure of microhelix RNA (μhRNA). **(B−E)** MALDI-TOF MS of μhRNA after flexizyme reaction and/or acetic anhydride reaction. Pro was charged on μhRNA by using a flexizyme variant, dFx. The same reaction conditions for the $tRNA^{ini}$ acylation/acetylation were applied. See the methods section for the details of reaction conditions. Calculated (calcd.) and observed (obsd.) *m/z* values of $[M+H]^+$ are indicated. Note that the average mass values are shown.
