# Supplementary material for: Drop-off-reinitiation at the amino termini of nascent peptides and its regulation by IF3, EF-G, and RRF
Source: RNA. 2023 May;29(5):663–74. doi: 10.1261/rna.079447.122 (PMC10158994; doi:10.1261/rna.079447.122)
Supplement: Supplemental Material [file supp_079447.122_Supplemental_Fig_S3_.pdf]

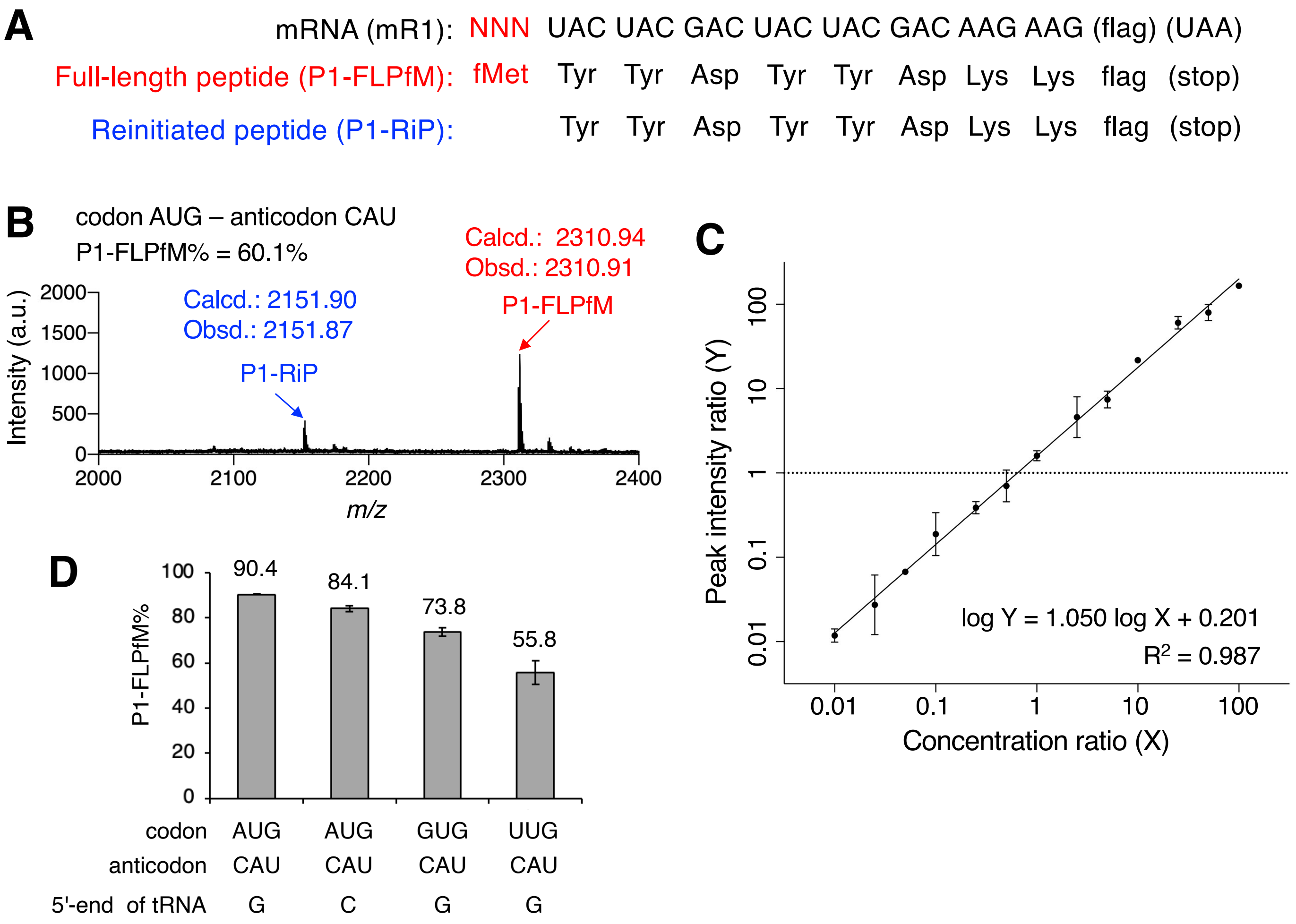

SUPPLEMENTAL FIGURE S3. In vitro translation of a model peptide introducing *N*-formyl-L-methionine at the amino-terminus. **(A)** Sequences of mRNA, mR1, and the corresponding peptide sequences, P1-FLPfM and P1-RiP. P1-FLPfM is a full-length peptide bearing *N*-formyl-L-methionine (fMet) at the amino-terminus. P1-RiP is a reinitiated peptide lacking the amino-terminal fMet. The amino acid sequence of 'flag' is Asp-Tyr-Lys-Asp-Asp-Asp-Asp-Lys, which is translated from GAC-UAC-AAG-GAC-GAC-GAC-GAC-AAG. **(B)** MALDI-TOF mass spectrum of the translated peptides. Translation was carried out in the absence of IF3 with 0.1 µM EF-G and 0.5 µM RRF. The amino-terminal fMet was introduced at the canonical initiation codon AUG using anticodon CAU. Red and blue arrows indicate P1-FLPfM and P1-RiP, respectively. Calculated (calcd.) and observed (obsd.) *m/z* values of $[M+H]^+$ are indicated. P1-FLPfM% was calculated using the regression line [2] shown in (C). **(C)** Correlation between peak intensity ratio and concentration ratio of P1-FLPfM to P1-RiP. Mixtures of known concentration ratios of P1-FLPfM and P1-RiP were analyzed by MALDI-TOF MS to determine their peak intensity ratios. The equation of the regression line [2] and its correlation coefficient are shown in right bottom. **(D)** Evaluation of P1-FLPfM% with variations of codon and 5'-end nucleotide of initiator tRNA. Translation was performed in the presence of 1.5 µM IF3, 0.1 µM EF-G, and 0.5 µM RRF. Error bars, S.D. (n = 3).
