# Supplementary material for: Drop-off-reinitiation at the amino termini of nascent peptides and its regulation by IF3, EF-G, and RRF
Source: RNA. 2023 May;29(5):663–74. doi: 10.1261/rna.079447.122 (PMC10158994; doi:10.1261/rna.079447.122)
Supplement: Supplemental Material [file supp_079447.122_Supplemental_Figure_S1.pdf]

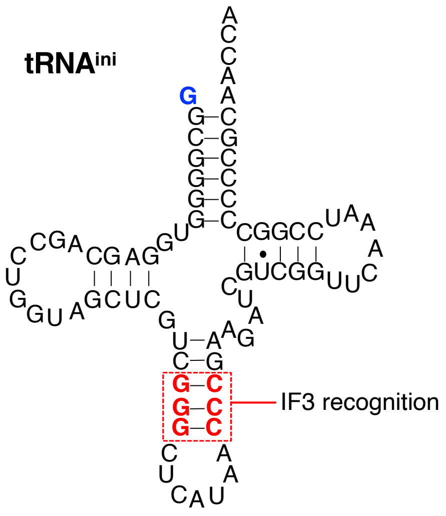

SUPPLEMENTAL FIGURE S1. Secondary structure of tRNA$^{ini}_{CAU}$ used for incorporation of AcPro or fMet at the N-terminus of P1 peptide. Note that the C at the 5' end of wild-type *E. coli* tRNA^{ini} is substituted with G to improve transcription yield (indicated by blue). Nucleotides required for IF3 recognition are indicated by red.
